# A Subset of Malignant Mesothelioma Tumors Retain Osteogenic Potential

**DOI:** 10.1038/srep36349

**Published:** 2016-11-25

**Authors:** S. M. Lansley, B. Pedersen, C. Robinson, R. G. Searles, G. Sterrett, I. van Bruggen, R. A. Lake, S. E. Mutsaers, C. M. Prêle

**Affiliations:** 1 Institute for Respiratory Health and Centre for Respiratory Health, School of Medicine and Pharmacology, University of Western Australia, Harry Perkins Institute of Medical Research, Nedlands, WA, Australia; 2 National Centre for Asbestos Related Disease, School of Medicine and Pharmacology, University of Western Australia, Nedlands, WA, Australia; 3 Harry Perkins Institute of Medical Research, Nedlands, WA, Australia; 4 Anatomical Pathology Research, PathWest Laboratory Medicine WA and School of Pathology, University of Western Australia, Nedlands, WA, Australia; 5 Centre for Cell Therapy and Regenerative Medicine, School of Medicine and Pharmacology, University of Western Australia and Harry Perkins Institute of Medical Research, Nedlands, WA, Australia

## Abstract

Malignant mesothelioma (MM) is an aggressive serosal tumor associated with asbestos exposure. We previously demonstrated that mesothelial cells differentiate into cells of different mesenchymal lineages and hypothesize that osseous tissue observed in a subset of MM patients is due to local differentiation of MM cells. In this study, the capacity of human and mouse MM cells to differentiate into osteoblast-like cells was determined *in vitro* using a functional model of bone nodule formation and *in vivo* using an established model of MM. Human and murine MM cell lines cultured in osteogenic medium expressed alkaline phosphatase and formed mineralized bone-like nodules. Several human and mouse MM cell lines also expressed a number of osteoblast phenotype markers, including runt-related transcription factor 2 (RUNX2), osteopontin, osteonectin and bone sialoprotein mRNA and protein. Histological analysis of murine MM tumors identified areas of ossification within the tumor, similar to those observed in human MM biopsies. These data demonstrate the ability of MM to differentiate into another mesenchymal cell type and suggest that MM cells may contribute to the formation of the heterologous elements observed in MM tumors.

Malignant mesothelioma (MM) is a rare but aggressive primary tumor of the serosa associated with past exposure to asbestos^1^. MM is typically subdivided into three histological subsets: epithelioid, sarcomatoid and biphasic (contains both components); the sarcomatous subtype is associated with a worse prognostic outcome^2^. Furthermore, MM has also been reported to contain areas of mesenchymal differentiation, including osseous and cartilaginous differentiation^3^,^4^,^5^ which is classified as a further subset of the sarcomatous type of MM^6^.

It has been proposed that the mesenchymal components (osseous and cartilaginous) and the variability of the histological subtypes of MM are due to the capacity of mesothelial-derived cells to differentiate into multiple cell lineages of the embryonic mesoderm (termed multipotent)^7^. In the early 19^th^ century, Durante and Conheim presented the embryonal rest theory of cancer, which stated that remnants of embryonic tissue remain in adult organs and that a change in the surrounding environment would allow the embryonic tissue to proliferate and produce masses of cells that resemble fetal tissues^8^. This is now known as the stem cell theory of cancer. Indeed, Donna and Provana^7^ introduced the term ‘mesodermoma’ to classify primary tumors of the serous surfaces by considering the embryological development of serous membranes as being derived from the multipotent mesoderm. Recently, MM cells with cancer stem cell characteristics have been identified^9^,^10^,^11^,^12^,^13^,^14^. We and others have recently shown that normal mesothelial cells express some of the markers associated with the mesenchymal lineage and can differentiate to an osteoblast phenotype *in vitro* under the influence of osteogenic medium (OM)^4^,^15^. Given the evidence of osteoid elements within MM biopsy samples, we hypothesize that MM cells have plasticity and are capable of differentiating into osteoblast-like cells and thus may be responsible for the elements of differentiated bone tissue observed in a subset of MM patients.

## Results

### Evidence of differentiated bone tissue in human and murine malignant mesothelioma tumors

**Figure 1:**
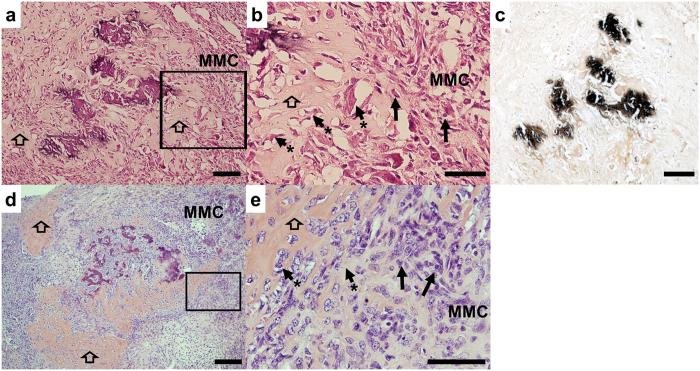
Identification of bone tissue within malignant mesothelioma. (**a)** H&E stained biopsy from a patient with pleural MM demonstrating malignant mesothelioma cells (MMC) and a central foci of bone tissue (dark stained) and osteoid (indicated by open arrowhead), scale bar = 200 μm. (**b)** Higher magnification of boxed area in a, highlighting that cells within the osteoid (arrowhead*) area are spindle-shaped neoplastic cells, similar to MMC (black arrows right section). (**c)** von Kossa staining demonstrating mineralization. (**b**,**c**) Scale bar = 200 μm. (**d)** Bone tissue (dark stained) and osteoid (open arrowhead) in murine wild-type MM tumor tissue surrounded by MMC. Scale bar = 200 μm. (**e**) Higher magnification of boxed area in (**d**), showing that tumor cells on the right of the section (black arrow) resemble those forming osteoid on the left (arrowhead*). Scale bar = 200 μm.

Analysis of a MM biopsy from a 27 year-old woman with childhood exposure to asbestos showed areas of mineralized bone formation within the tumor (Fig. 1a,b) which was confirmed using von Kossa staining (Fig. 1c). Areas of calcified osteoid (eosinophilic background) surrounded by cellular sarcomatoid tissue is shown (Fig. 1a). Higher magnification showed that the spindle shaped cells located within the osteoid area had morphological malignant characteristics that closely resembled cells in the adjacent sarcomatoid mesothelioma tissue (Fig. 1b), supporting the notion that these cells originate from a common precursor. These malignant characteristics included nuclear enlargement, irregular nuclear size and shape and cells with a high nuclear to cytoplasmic ratio.

Similar areas of bone tissue were identified in C57BL/6 MM tumors (Fig. 1d,e). Murine MM tumors were obtained from 50 mice on a C57BL/6 background following asbestos exposure. Out of the 50 mice analyzed, 21% of MexTAg and 27.2% of C57BL/6 wild-type tumor samples contained bone. The murine tumors demonstrated a pure sarcomatous growth pattern with no epithelial elements (Fig. 1e). There appeared to be a progression from sarcomatoid tumor with a “fibroblastic” appearance (Fig. 1e) through to tumor producing abundant eosinophilic osteoid but containing pleomorphic, malignant tumor cells, similar to those in the fibroblastic foci (Fig. 1e).

### Differential alkaline phosphatase (AP) expression and mineral deposition in human and murine MM cells

**Figure 2:**
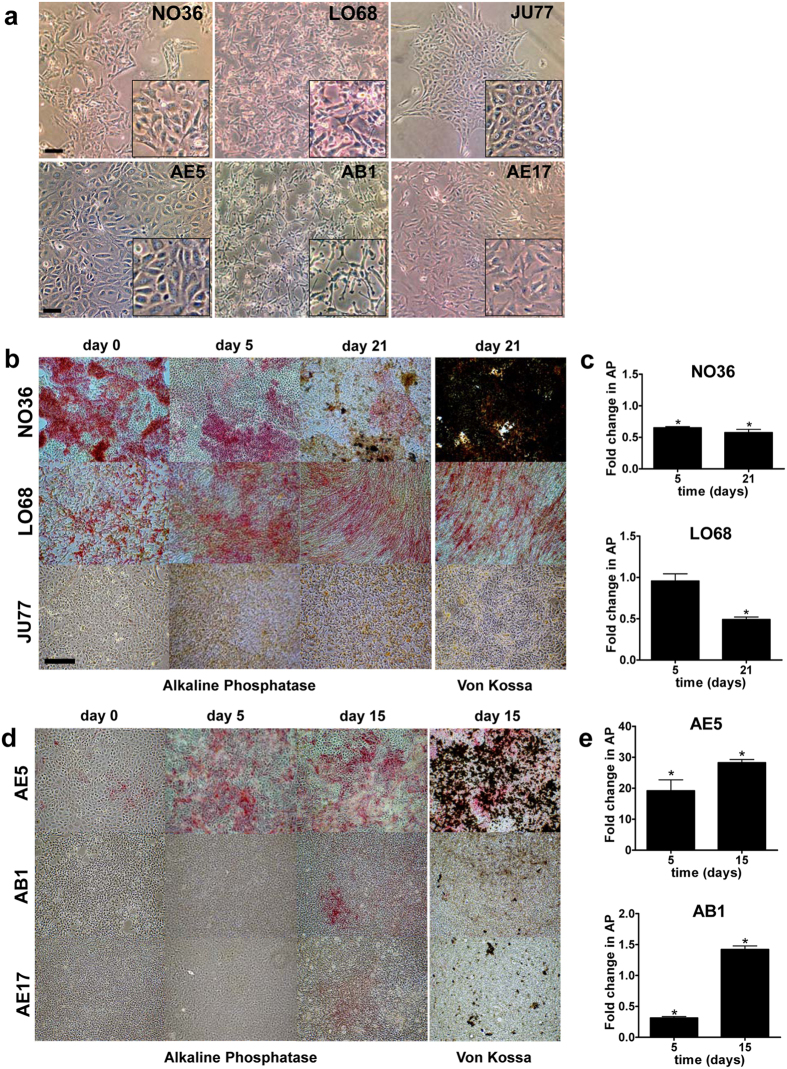
Human and mouse malignant mesothelioma cells have osteogenic potential *in vitro*. The osteogenic potential of three human (NO36, LO68 and JU77) and three murine (AE5, AB1 and AE17) MM cell cultures was determined using an *in vitro* assay of bone nodule formation and mineralization. Cells were cultured for up to 21 days in the presence of osteogenic medium. (**a)** Under standard culture conditions at day 0, the three human MM cell lines displayed different morphologies. NO36 and JU77 displayed an epithelioid morphology compared to the fibroblastic or spindle morphology of LO68. Scale bar = 200 μm (higher magnification insets are also shown). (**b)** NO36 cells express high levels of the osteoblast phenotype marker AP (pink staining), and demonstrate extensive mineralization as demonstrated by von Kossa staining (black areas) at day 21. Lower levels of AP were detected in LO68 and no AP was expressed by JU77. (**c)** Quantitative analysis of AP in protein lysates demonstrates a decrease in AP in LO68 over time. Data represented as fold change in AP expression compared to untreated ± SEM, n = 3 experiments p < 0.05. (**d)** High levels of AP and mineralization are seen in AE5 cultures. AB1 and AE17 express AP at a lower level. (**e)** AP expression increases over time in culture in AE5 and AB1 cells exposed to OM for up to 15 days. Data represented as fold change in AP expression compared to untreated ± SEM, n = 3 experiments, p < 0.05.

Three human (NO36, JU77 and LO68) and three mouse (AE5, AB1 and AE17) MM cell lines were examined. NO36, JU77 and AE5 cells displayed an epithelioid morphology whereas LO68, AB1 and AE17 cells exhibited a more spindle morphology (Fig. 2a). Following differentiation in osteogenic medium for up to 21 days, expression of AP was detected in two of the three human MM cell lines, NO36 and LO68 (Fig. 2b). Histologically, the NO36 cell line expressed higher levels of AP compared to LO68. Quantitative analysis of AP expression in NO36 and LO68 revealed that AP expression decreased with time in culture (Fig. 2c). AP expression also varied between murine MM cultures (Fig. 2d). AE5 cells expressed higher levels of AP compared to AB1 and AE17 cells (Fig. 2e). AP levels increased with time in AE5 and AB1 cultures in the presence of osteogenic medium. However the levels of expression and induction of AP remained higher in AE5 cultures (Fig. 2e). JU77 and AE17 AP levels were below detectable limits (data not shown).

Cell lines expressing high AP levels also stained more intensely with von Kossa, demonstrating the presence of mineralized nodules (Fig. 2b,d). AP levels decreased by day 5 in the NO36 cell line which corresponded with matrix maturation and mineral deposition. NO36 was the only human MM cell line to form mineralized nodules, whereas all three of the murine MM cell lines deposited mineral *in vitro* (black staining).

### MM cells express markers associated with the osteoblast lineage

**Figure 3:**
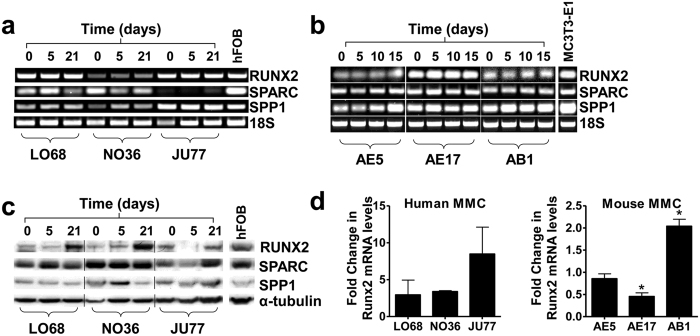
Malignant mesothelioma cells retain their osteogenic potential. (**a)** Human (LO68, NO36, JU77) and (**b)** mouse (AE5, AE17 and AB1) MM cells express the osteoblast phenotype markers RUNX2, SPARC and SPP1 mRNA at all time points examined. Human JU77 cells expressed low levels of SPARC mRNA at all time points. 18S ribosomal RNA is used as an endogenous control. Human foetal osteoblasts (hFOB) or mouse osteoblasts (MC3T3-E1) were used as positive controls for the osteoblast phenotype. (**c)** Human MM cells express RUNX2, SPARC and SPP1 protein. RUNX2 expression increased with time in culture. α-Tubulin is used as a lane loading control. (**d)** Quantitative analysis of bone-specific RUNX2 mRNA expression shows increased RUNX2 in OM-differentiated human MM at day 21 (p < 0.05). In contrast, the levels of bone-specific RUNX2 mRNA varied between murine MM cell lines. The results are representative of n = 3 (human) and n = 4 (murine) experiments for each cell line. Fold change was calculated as compared to Day 0 (untreated) for each cell line.

The pathway leading to bone formation has been well described^16^. Malignant mesothelioma cells were screened for the expression of markers associated with the osteoblast cell lineage; RUNX2, SPARC and SPP1 (Fig. 3).

All MM cell lines analyzed expressed RUNX2, SPARC and SPP1 mRNA and protein at day 0 and throughout the nodule formation assay (Fig. 3a,d). The protein expression of RUNX2, a protein specifically associated with the mature osteoblast phenotype, increased at day 21 in each of the human MM cultures (Fig. 3c) with no apparent change in the expression of SPP1 and SPARC. Quantitative PCR analysis of bone-specific RUNX2 in human MM cells revealed increased levels of RUNX2 mRNA at day 21 (Fig. 3d) when compared to day 0 (fold-change). In contrast, only one mouse cell line (AB1) displayed elevated levels of RUNX2 at day 15 (Fig. 3d).

### MM cells produce soluble factors that stimulate AP expression *in vitro*

**Figure 4:**
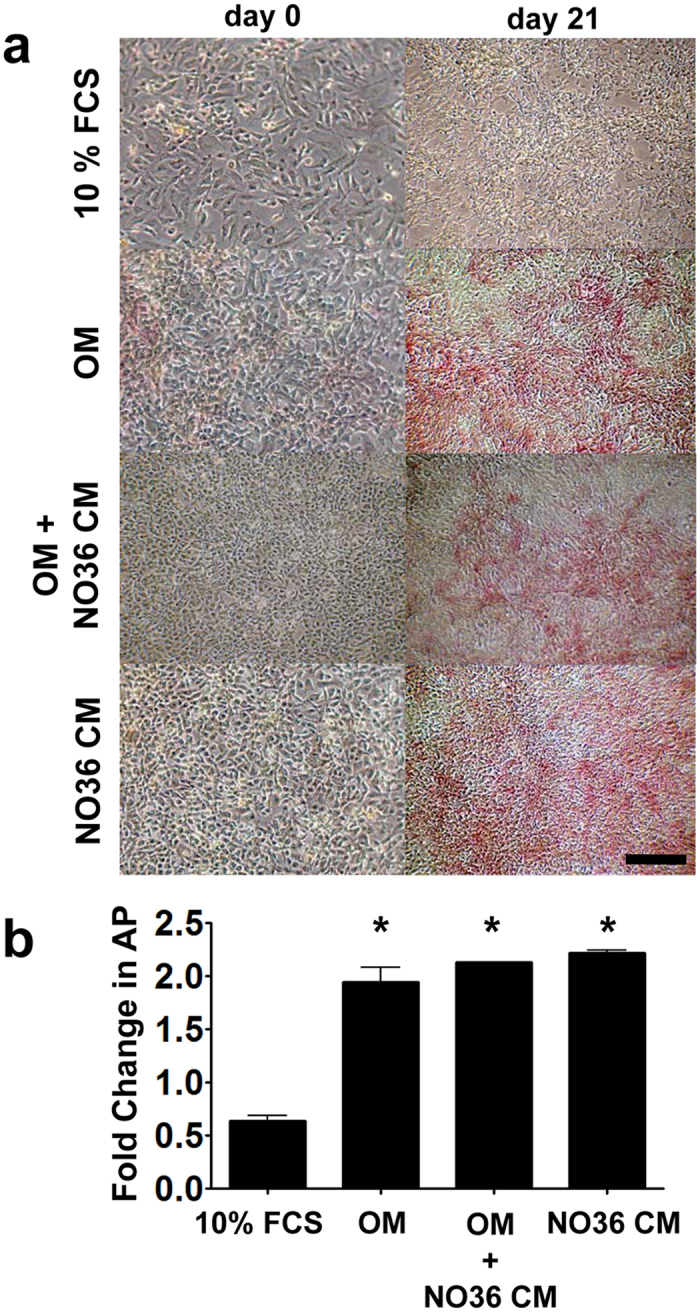
Conditioned medium from NO36 cells is sufficient to drive osteogenic differentiation but not mineralization. LO68 cells were incubated either under standard culture conditions (10% FCS), in the presence of OM, conditioned medium harvested from confluent NO36 cultures (at 24 hours; NO36 CM) or OM + NO36 CM for 21 days. (**a)** AP staining demonstrated both OM and NO36 CM can induce AP expression in LO68 cells. (**b)** Quantitative analysis of AP expression in protein lysates harvested at day 21 confirmed that NO36 CM had the same capacity to induce osteoblast differentiation in human MM as OM alone. No mineralization was detected in these cultures. Scale Bar = 200 μm. There was no significant difference in AP expression levels between OM and NO36 CM treated cultures at day 21. However, all OM containing cultures demonstrated significantly higher AP expression compared to 10% FCS controls at day 21 (*p < 0.05).

To determine if NO36 cells produce a soluble factor that augments osteoblast differentiation *in vitro*, LO68 cells, which do not express high levels of alkaline phosphatase or mineralize when cultured in OM, were grown to confluence and incubated in NO36 conditioned medium (NO36 CM), OM, OM + NO36 CM or 10%FCS medium for 21 days. NO36 CM stimulated similar levels of AP production in LO68 cells compared with OM and OM + NO36 CM (Fig. 4a,b), suggesting that a soluble factor/s present in NO36 CM could be driving differentiation. LO68 cells did not form mineralized nodules in any of the treatment groups consistent with previous findings for OM treatment (Fig. 2 and not shown).

### Dexamethasone (DEX) treatment of tumor bearing mice does not enhance osteogenic differentiation of MM tumors *in vivo*

**Figure 5:**
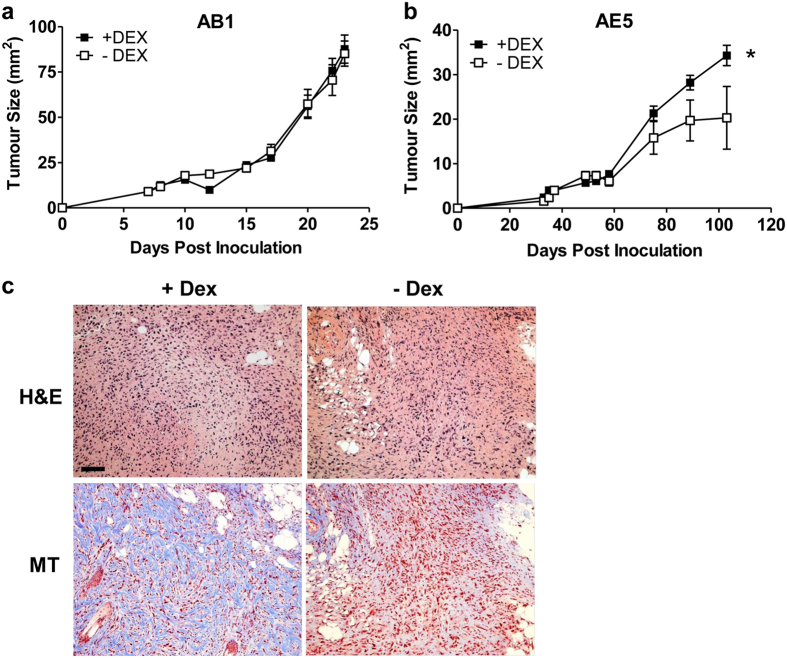
Dexamethasone treatment of tumor bearing mice does not enhance osteogenic differentiation but selectively affects tumor growth *in vivo.* Mice were injected with either AE5 or AB1 syngeneic MM cells into the hind flank and tumor growth rates in the presence or absence of DEX determined over time. Data represents tumor size in 10 mice ± SEM. AE5 tumors treated with DEX demonstrated a significant increase in tumor burden at cessation of treatment ((**b**) *p<0.05). There was no difference in tumor size in AB1 DEX treated tumors compared with controls (**a**). Histological analysis did not identify areas of bone formation in either AB1 or AE5 tumors but the latter demonstrated extensive dermal invasion with abundant collagen deposition ((**c**) MT). Scale bar = 100 μm.

DEX is commonly used in MM patients as an antiemetic or as prophylactic treatment for skin rashes that develop as a result of chemotherapy^17^. DEX is also used *in vitro* to induce osteogenic differentiation of a number of different cell types^18^,^19^. Thus, we hypothesized that exposure to DEX may drive tumor differentiation *in vivo*. To test this hypothesis mice were inoculated with either the AE5 (AP high expression) or AB1 (AP intermediate expression) syngeneic murine MM cells subcutaneously into the hind flank of C57BL/6 or BALB/c mice respectively. AB1 tumors followed a similar pattern of growth in both treatment and control groups (Fig. 5a). However, a significant increase in tumor growth was observed in DEX-treated, AE5 inoculated animals compared to saline controls at end point (100 days) (p < 0.0001; Fig. 5b). There were no overt histological differences in the appearance of MM cells within DEX or saline treated AB1 tumors (data not shown). DEX treatment did not enhance bone formation in AE5 or AB1 MM tumors. However, tumor invasion into the dermis was observed and Masson’s trichrome (MT) staining demonstrated abundant collagen in DEX-treated AE5 tumors but not saline treated controls (Fig. 5c).

## Discussion

Over 40 cases of MM with heterologous elements have been described in the literature^3^,^4^,^5^ but the origin of these unusual variants has remained elusive. We have previously demonstrated that normal mesothelial cells can differentiate to an osteogenic phenotype^4^ and therefore hypothesize that all cells of mesothelial origin, including MM, are responsible for the observed osseous differentiation and mineralized bone formation in MM tumors.

Tumor biopsy samples from a patient with pleural MM and MM tumors derived from mice showed bone tissue. Histologically, the osteoid tissue in the murine MM tumors closely resembled the human MM tumor indicating that osseous differentiation within the tumors is likely to be derived from the MM tumor cells and not from metastatic disease as has been previously proposed^20^. Osseous differentiation from other possible precursor cells within the tumour cannot be excluded, but data presented in this study provides strong support for a MM cell origin.

Putative cancer stem cells have been identified in many different tumor types including breast and prostate cancer, glioblastoma, neuroblastoma, mesenchymal chondrosarcomas, metastatic osteosarcomas and MM^21^,^22^,^23^. Many have shown a capacity for multilineage differentiation into tumor-supporting vascular and stromal cells and endothelium^24^,^25^,^26^,^27^. The current study is the first to demonstrate a capacity for mesothelioma tumor cell differentiation to the osteoblast lineage, providing further evidence for a mesothelial progenitor origin for the osseous elements found within a subset of mesothelioma tumors.

There are many reports in the literature demonstrating the capacity of mesothelial cells to undergo epithelial to mesenchymal transition (EMT), or what has more recently been described as mesothelial to mesenchymal transition (MMT), and differentiate into cells of different phenotypes^4^,^28^,^29^,^30^,^31^. During development, mesothelium lining the heart, liver, lungs and gut differentiate into vascular smooth muscle cells, interstitial fibroblasts, hepatic stellate cells, endothelial cells and adipocytes^32^,^33^,^34^,^35^,^36^,^37^. More recently, adult mesothelial cells have also been shown to undergo MMT and differentiate into vascular smooth muscle cells, adipocytes and osteoblasts^4^,^38^,^39^ and are likely to play important roles in fibrogenesis^40^. However, whether MM cells retain the potential to differentiate into other malignant or benign cell types is unknown.

In the current study, we used MM cell lines of different histological subtypes to examine their ability to differentiate into osteoblasts under appropriate culture conditions. We had hypothesized that the mesenchymal or sarcomatoid phenotype would have the greatest capacity for differentiation as cells generally differentiate into a mesenchymal phenotype before differentiating into osteoid cells. Interestingly, although each MM cell line demonstrated a capacity for osteoblast-like differentiation, epithelioid MM cells had the greatest capacity to differentiate. Whether or not the better prognosis associated with the epithelioid MM phenotype^3^, can be attributed to the ability of these cells to differentiate into bone requires further investigation.

We clearly show mRNA and protein expression of several key osteoblast markers. Interestingly, bone-specific RUNX2 was expressed in unstimulated (day 0) MM cells, consistent with our observation in normal mesothelial cells^4^. We previously suggested that this may be a legacy of their embryonic mesodermal origin and therefore these cells retain the potential of the embryonic mesoderm for differentiation to diverse cell lineages in a manner similar to that of mesenchymal stem cells. The variability in AP, RUNX2, SPARC and SPP1 expression and the disparate capacity for differentiation observed in the human and murine MM cell lines is also consistent with clinical observations, as not all patients with MM are reported to contain areas of osseous differentiation and bone formation. However, a true account of the number of MM cases containing bone is unclear due to the small amount of biopsy tissue collected for diagnosis and the fact that many mesotheliomas are diagnosed solely by pleural effusion cytology^41^.

It is unclear what drives the osteoblast differentiation within the tumor. It has been suggested that a soluble mediator, perhaps induced or produced by MM cells, stimulates the formation of bone^20^. The NO36 cell line demonstrated the greatest capacity for osteogenic differentiation and therefore we investigated whether factors produced by NO36 cells could induce osteogenic differentiation of other MM cell lines. NO36-CM induced AP expression in LO68 cells but did not induce formation of mineralized nodules, suggesting that other factors may be required to drive mineralization. MM cells express several bone-related factors which may be involved in osseous differentiation including bone morphogenetic protein-2^42^, transforming growth factor beta^43^ and Sonic hedgehog^44^. Further studies are needed to determine which factor(s) produced by MM cells or surrounding tumor stroma are required to drive MM differentiation.

Dexamethasone (DEX) is known to induce the osteogenic differentiation of pre-osteoblasts, mesenchymal stem cells and embryonic stem cells at physiological levels^45^,^46^,^47^. DEX is also often used to treat inflammation in MM patients. Therefore we examined the correlation between DEX treatment and bone formation using a murine model of MM. AE5 murine MM cells displayed a greater capacity for osteogenesis and mineralization *in vitro* compared with the other two cell lines examined and AB1 cells demonstrated a lower differentiation capacity *in vitro*. Therefore the effect of DEX treatment on the growth and osteoid formation of these cells were compared *in vivo*. DEX treatment did not induce bone formation in either cell line, however tumor growth was significantly increased in DEX treated AE5 cells with evidence of dermal invasion and abundant collagen. DEX has been shown to stimulate connective tissue growth factor expression in mouse fibroblasts which leads to increased collagen production^48^ which may explain the increase in connective tissue within DEX treated tumors. Although these findings are interesting and suggest a possible detrimental effect of DEX treatment in some MM patients, further studies are needed to confirm this response and determine its mechanism of action and how this may affect clinical outcome.

Exactly how the unusual histological variants (heterologous elements) of MM may affect patient prognosis is currently unknown. Demirag and colleagues^6^ have described two MM cases where patients presenting with biphasic MM and osseous differentiation have survived up to 69 months post diagnosis, compared to the 18 months usually documented^6^. Recently, Klebe and colleagues^3^ described the clinical characteristics and prognosis of 27 cases of MM with heterologous elements which predominately consisted of osteosarcomatous and chondrosarcomatous constituents. In their series, the incidence of MM with these elements corresponded to 0.05% of all MM cases. The authors described a median survival of 6 months after diagnosis and one confirmed one-year survivor within this series and concluded that the slightly longer survival (6 versus 5.5 months^3^) may be attributed to the presence of epithelial elements in the biphasic MM cases analyzed. Survival data was not available for the patient studied here. A more detailed survival study using a large cohort of MM patients with and without bone formation is required to address this question. However, if a positive correlation between heterologous elements in MM and patient survival exists, inducing MM cells to differentiate into non-malignant phenotypes *in situ* may be a treatment option. Differentiation therapy has already been used in several different cancers to induce terminal differentiation in cancer stem cells to decrease cell proliferation and halt tumor progression^49^.

This study provides further evidence for the multipotent nature of mesothelial-derived cells^4^,^7^ and use for the classification of ‘mesodermoma’^7^ and heterologous elements^3^. These data provide a strong basis for additional studies to better understand the origin of the unusual histological variants observed in MM and to investigate their relation to tumor progression and survival outcomes.

## Materials and Methods

### Cell culture

Three previously established human (NO36, LO68 and JU77) MM cell lines isolated from pleural effusions of MM patients^50^ and three murine (AE5, AB1 and AE17) MM cell lines derived from mice inoculated with asbestos^51^,^52^ were used in this study. The murine osteoblast cell line, MC3T3 E1 and the human fetal osteoblast cell line, hFOB 1.19 were obtained from the American Type Culture Collection (ATCC) and used as positive controls for the osteoblast phenotype. All cells were maintained in standard medium containing DMEM with high glucose (4.5 mg/L) supplemented with 10% foetal calf serum (FCS), 4 mM L-glutamine, 100,000 units/L penicillin and 50 mg/mL streptomycin (Invitrogen Life Technologies, Mulgrave, Victoria, Australia).

### *In vitro* bone nodule formation assay

Human and murine MM cells were maintained in either standard medium (DMEM with high glucose (4.5 mg/L) supplemented with 10% foetal calf serum (FCS), 4 mM L-glutamine, 100,000 units/L penicillin and 50 mg/mL streptomycin) or osteogenic medium (OM^4^) for up to 21 days. For NO36 conditioned medium (NO36 CM) experiments using LO68 cells, the following culture conditions were used; LO68 cells were either cultured in 2 mL of standard medium, OM or NO36 CM for up to 21 days. NO36 CM was derived by harvesting supernatants from confluent NO36 cells cultured under standard conditions for 24 hours. Cell differentiation into osteoblast-like cells was analyzed at multiple time points throughout the study. Cells were stained histochemically for alkaline phosphatase expression and/or von Kossa as previously described^4^. Quantitative analysis of alkaline phosphatase expression was determined in protein extracts using a colometric assay^4^.

### Reverse transcription PCR (RT-PCR) and real-time RT-PCR

Total RNA was isolated from OM-differentiated murine and human MM cell cultures at several time points using Trizol reagent (Invitrogen Life Technologies) as per the manufacturer’s instructions and DNase treated prior to cDNA synthesis (Invitrogen Life Technologies). cDNA was synthesized using a Superscript III first strand synthesis kit (Invitrogen Life Technologies). The mRNA expression of human osteoblast phenotype markers was determined using gene specific primers and standard PCR conditions as previously reported^4^. The murine gene specific primers used in this study are outlined in Table 1. PCR reactions were carried out at 95 °C for 45 seconds, 55 °C for 45 seconds, and 72 °C for 45 seconds for 35 cycles and PCR products visualized on a 1% agarose gel stained with ethidium bromide. Real Time PCR analysis of runt-related transcription factor 2 (RUNX2) mRNA was performed using Taqman probes on an Applied Biosystems 7300 real-time PCR ((Applied Biosystems); Table 2). Taqman Real Time PCR reactions were carried out at 95 °C for 15 seconds and 60 °C for 1 min for 40 cycles. Gene expression was normalised to 18S ribosomal RNA. Fold change was determined by comparison to day 0 (untreated controls) of the same cell line.

### Western blot analysis

Cells were lysed in breaking buffer as previously described^4^. Approximately 30 μg of protein was resolved per lane of a 4–20% precast SDS-PAGE NuPAGE^®^ Novex^®^ bis-tris gel (Invitrogen Life Technologies) and protein immobilized on a PVDF membrane (Amersham, Buckingham Shire, UK). Membranes were blocked overnight at 4 °C in 5% skim milk/TBS-T (TBS with 0.05% Tween 20). Following serial washing the membranes were incubated with either mouse RUNX2 (Santa Cruz Biotechnology, Santa Cruz, CA), rat secreted protein, acidic, cysteine rich (SPARC; Developmental Studies Hybridoma Bank, University of Iowa), rat secreted phosphoprotein 1 (SPP1; Abcam, Cambridge, UK) or anti-α-tubulin (Sigma Aldrich, NSW, Australia). Membranes were washed and incubated with HRP-conjugated anti-rabbit (Pierce Biotechnology, IL) or anti-mouse antibody (DakoCytomation, Carpinteria, CA). Membranes were visualized using the chemiluminescent peroxidase substrate (CPS-1-120, Sigma Aldrich) and hyperfilm ECL (GE Healthcare, UK).

### *In vivo* differentiation assay

All animal experiments were undertaken with the approval of the Animal Ethics Committee of the University of Western Australia (RA/05/100/505) in accordance with National Health and Medical Research Committee guidelines and regulations.

Approximately, 2 × 10^6^ AB1 and 3.5 × 10^6^ AE5 syngeneic cells were injected subcutaneously into the right flank of either BALB/C (AB1) or C57BL/6 (AE5) mice respectively and tumor growth monitored by taking perpendicular measurements using microcalipers. Once tumors reached approximately 1 mm^2^, 10 animals per group were treated with either dexamethasone (DEX; 0.5 mg/kg) or saline via intra-peritoneal injection for 22 days. Once tumors had either reached 100 mm^2^ or had grown for a pre-determined amount of time they were removed and paraffin embedded for histological evaluation.

### Histopathology

All human samples were obtained and used in accordance with National Health and Medical Research Committee guidelines and regulations and ethics approved by the Sir Charles Gairdner Ethics Committee.

Formalin-fixed, paraffin-embedded (FFPE) lung tissue sections from a 27 year old female patient with a confirmed diagnosis of malignant mesothelioma were obtained from retrospective diagnostic biopsy specimens from PathWest Laboratory Medicine (Perth, Western Australia). Analysis of murine tumors was performed on archival tumor tissue samples obtained from 39 MexTAg mice and 11 C57BL/6 wild-type (WT) mice inoculated with asbestos as described^53^ and fresh tumor tissue samples obtained from mice treated with dexamethasone. Histological analysis of haematoxylin and eosin (H&E), Masson’s trichrome (MT; collagen) and von Kossa (mineralization) stained sections was confirmed by a senior pathologist (PathWest Laboratory Medicine).

### Statistical Analysis

All data are presented as mean ± standard error of mean (SEM). Comparisons between individual time points were performed using a one sample t-test or a one way ANOVA using a Tukey’s Multiple Comparison test as appropriate to determine significance. A p value of less than 0.05 was considered significant.

## Additional Information

**How to cite this article**: Lansley, S. M. *et al*. A Subset of Malignant Mesothelioma Tumors Retain Osteogenic Potential. *Sci. Rep.* **6**, 36349; doi: 10.1038/srep36349 (2016).

**Publisher’s note:** Springer Nature remains neutral with regard to jurisdictional claims in published maps and institutional affiliations.

## Figures and Tables

**Table 1:** Murine Primers used for RT-PCR.

Primer Name		Sequence 5′-3′
SPP1	Forward	TAAGCAAGAAACTCTTCCAA
	Reverse	CATCAGGATACTGTTCATCA
SPARC	Forward	CATTACGCAGGATGCTCAGA
	Reverse	TAGGAATGCCCATCAGAACC
18S	Forward	TCGAACGTCTGCCCTATCAA
	Reverse	GCTATTGGAGCTGGAATTACCG

**Table 2:** Primers and probes designed for Real Time PCR of RUNX2.

Species	Primer Name		Sequence 5′-3′
Human	RUNX2	Forward	GCACCAAGTCCTTTTAATCCACAA
		Reverse	GGGAAGACTGTGCCTGCCT
		Probe	TCAGATTACAGACCCC
Murine	RUNX2	Forward	CAAGAAGGCTCTGGCGTTTAA
		Reverse	TACTGCTTGCAGCCTTAAATGACT
		Probe	CAGGTCACTACCAGCCA
